# Identification of *F5* as a Prognostic Biomarker in Patients with Gastric Cancer

**DOI:** 10.1155/2020/9280841

**Published:** 2020-02-27

**Authors:** Yi Liu, Xi-Wen Liao, Yu-Zhou Qin, Xian-Wei Mo, Shan-Shan Luo

**Affiliations:** ^1^Department of Gastrointestinal Surgery, Guangxi Medical University Cancer Hospital, Guangxi Clinical Research Center for Colorectal Cancer, Nanning, 530021 Guangxi Zhuang Autonomous Region, China; ^2^Department of Hepatobiliary Surgery, The First Affiliated Hospital of Guangxi Medical University, Nanning, 530021 Guangxi Zhuang Autonomous Region, China

## Abstract

Association of Coagulation factor V (*F5*) polymorphisms with the occurrence of many types of cancers has been widely reported, but whether it is of prognostic relevance in some cancers remain to be resolved. The RNA-sequencing dataset was downloaded from The Cancer Genome Atlas (TCGA). The potential of *F5* genes to predict the survival time of gastric cancer (GC) patients was investigated using univariate and multivariate survival analysis whereas “Kaplan-Meier plotter” (KM-plotter) online tools were employed to validate the outcomes. TCGA data revealed that *F5 mRNA* levels were significantly upregulated in gastric cancer samples. Survival analysis confirmed that high levels of *F5 mRNA* correlated with short overall survival (OS) in gastric cancer patients, and the area under the curve (AUC) values of 1-, 2-, and 5-year OS rate were 0.554, 0.593, and 0.603, respectively. Survival analysis by KM-plotter indicated that the high expression of *F5 mRNA* was significantly associated with a shorter OS compared with the low expression level in all patients with GC, and this was also the case for patients in stage III (hazard ratio (HR) = 1.78, *P* = 0.017). These findings suggest that the *F5* gene is significantly upregulated in GC tumour tissues, and may be a potential prognostic biomarker for GC.

## 1. Introduction

Gastric cancer is the 5th most common malignancy globally and is the 3rd most lethal among all cancers; its incidence varies across regions [1]. Despite recent advances in the diagnosis and treatment methods of cancer, stomach cancer remains the 2nd leading cause of death among all cancers in China [2]. Moreover, it has inconsistent therapeutic response and prognosis at various stages because of high tumour heterogeneity. Investigation of the molecular mechanism of cancer invasion, metastasis, occurrence, and prognosis from a genomics perspective, which might provide highly sensitive treatment approaches, is therefore highly desirable. This may lead to the identification of new prognostic and diagnostic indicators and therapeutic targets.

*F5* (Coagulation factor V) is a circulating high molecular weight (330 kDa) procofactor which plays a role in the blood coagulation cascade. When activated, it functions as a cofactor that activates coagulation factor X to convert prothrombin to thrombin [3]. In malignancy, activation of coagulation and fibrinolysis is frequently detected. Increasing evidence has indicated that activation of the coagulation system is associated with a higher risk of invasion, metastases, and eventually, worse outcome. In other words, activation of the coagulation system is beneficial to tumour development [4]. Miller et al. found that long-term activation of the coagulation pathway promotes tumourigenesis in the digestive tract in males [5]. Numerous studies have investigated the association of the *F5* polymorphism with the risk of multiple cancers, such as colorectal cancer and gastric cancer [6]. So far, fewer studies have investigated the relationship between *F5* and cancer prognosis, including gastric cancer, in which changes in the coagulation-fibrinolytic system are often present. The association of the *F5* expression level with the survival time of patients with basal breast cancer has been reported by a single study by Tinholt et al. [7]. Accumulating evidence suggests that various clotting system factors may influence the prognosis of cancer patients [4, 8–12]. However, the role and mechanism of *F5* gene expression in the prognosis of cancers are still unknown.

The Cancer Genome Atlas (TCGA) database contains molecular data of more than 20,000 primary cancers and paired adjacent samples covering 33 cancer types. An RNA-sequencing (RNA-Seq) dataset from TCGA was used to determine the impact potential of the *F5* gene to predict the prognosis of GC patients. The “Kaplan-Meier plotter” database, which contains the gene expression profile of 1,400 GC patients and follow-up information from the Gene Expression Omnibus (GEO) were used to validate the results obtained from TCGA database.

## 2. Materials and Methods

### 2.1. Survival Analysis and Diagnostic Value of the *F5* Gene

The RNA-Seq count dataset and matched clinical information of stomach adenocarcinoma (STAD) were downloaded from TCGA (https://portal.gdc.cancer.gov/; December 15, 2018) [13], and then the RNA-Seq count dataset was normalized by the DESeq package [14]. The data of GC patients was subcategorized to two groups based on the median of the gene expression level. The prognostic value of the *F5* expression and the clinical characteristics were initially evaluated using the logrank test and univariate Cox regression model to identify significant prognostic factors for GC. Multivariate Cox regression was performed to confirm the role of genes by adjusting for age, TNM stage, radiation therapy, targeted molecular therapy, and residual tumour. Subsequently, stratified analysis was conducted based on the clinical parameters to further investigate the relationship between differential expression of this gene and clinical parameters by univariate and multivariate analyses. Lastly, the KM-plotter database (http://kmplot.com/private/; June 15, 2019), which automatically subgroups the submitted gene into high- and low-risk groups by the median value, was used to generate KM survival plots for the *F5* gene with multiple clinical parameters such as stage TNM, Lauren classification, differentiation, and HER2 status. Next, a survival risk map was plotted and a receiver operating characteristic (ROC) curve analyses were performed to evaluate the accuracy of *F5* in distinguishing adjacent or tumour tissues. The prediction accuracy of the *F5* was determined by *survivalROC* in R software.

### 2.2. Comprehensive Analysis of Genome-Wide Coexpression of *F5*

To further explore the expression-regulation relationship between *F5* gene and other genes, genome-wide coexpression analysis was carried out using *corrplot* package in the R 3.5.1 platform. Coexpression relationships were deemed significant using the standard *P* value < 0.05 and |*r*| > 0.2 as the cut-off values. Subsequently, the DAVID (https://david.ncifcrf.gov/home.jsp) database was used for Gene Ontology (GO) and Kyoto Encyclopedia of Genes and Genomes (KEGG) enrichment analyses to determine the biological processes associated with these genes [15].

### 2.3. GSEA Analysis

The data were categorised into high- and low-expression groups based on the median expression level of *F5* gene and GSEA (http://www.broadinstitute.org/gsea/index.jsp) was then performed to explore the potential biological mechanisms that underlie the prognostic role of *F5* [16, 17]. c2(v6.2) and c5(v6.2), obtained from a gene set database, were used to investigate the biological processes in patients with high or low expression of *F5* genes. Biological processes were considered to be significantly enriched if they met a threshold of normalized *P* < 0.05.

### 2.4. Statistical Analysis

Logrank test was used to calculate OS and the *P* values for clinical characteristics including *F5* gene. Factors with *P* value < 0.05 were included in the multivariate Cox regression analysis. Hazard ratio (HR) and 95% confidence intervals (CI) were calculated to determine the relative risk for different influencing factors. All statistical analyses were performed using SPSS v.22.0 software (IBM Corp, Armonk, NY, USA) and R3.5.1. *P* < 0.05 was considered statistically significant.

## 3. Results

### 3.1. The Expression Level and Diagnostic Value of *F5* in Gastric Cancer

**Figure 1:**
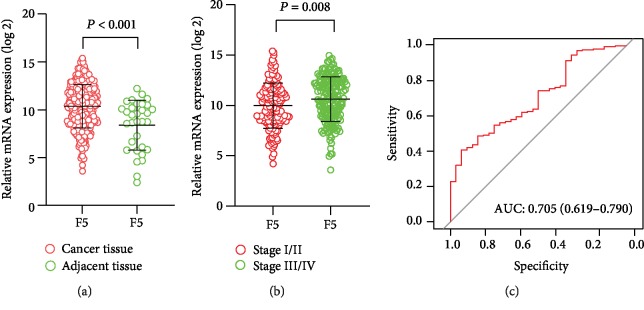
Distribution of *F5* genes in different gastric cancer tissues and tumour stages. The receiver operating characteristic curves (ROC) of the *F5* gene were used to distinguish gastric cancer tissue from normal tissues in The Cancer Genome Atlas (TCGA). (a) Gene expression of the *F5* gene in cancer tissues and adjacent tissues; (b) *F5* gene expression in different stages of gastric cancer; (c) receiver operating characteristic curves (ROC) of *F5* were used to distinguish gastric cancer tissue from adjacent normal tissues. *F5*: Coagulation factor V.

The RNA-Seq dataset of 351 GC patients from TCGA were included in current study. The distribution of *F5* gene differed significantly between adjacent tissues and tumour tissues, as well as in different stages as revealed by the scatter diagram (Figures 1(a) and 1(b)). The ability of *F5* to distinguish adjacent tissues from tumour tissues was medium (AUC (95%CI) = 0.705 (0.619−0.790) and Figure 1(c)).

### 3.2. Survival Analyses Using *F5* Gene

**Figure 2:**
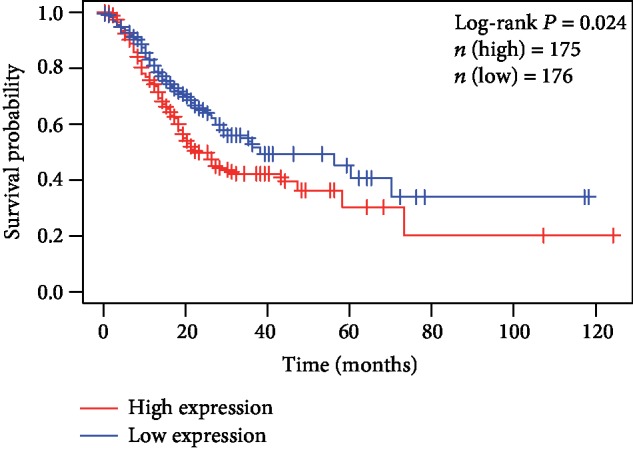
Kaplan-Meier survival curves of the *F5* gene in gastric cancer datasets from The Cancer Genome Atlas (TCGA).

**Figure 3:**
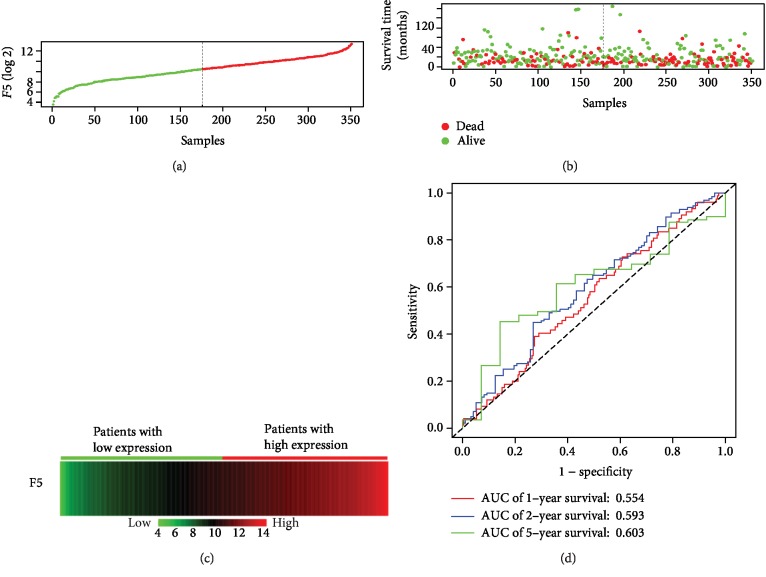
Prognostic value of *F5* in patients' dataset from TCGA cohort (*n* = 351). (a) *F5* gene distribution; (b) patient overall survival status and survival time. The dotted line divides the patients into low-expression and high-expression groups based on median gene expression. (c) Gene expression heat map of the *F5* expression profiles. Rows represent the *F5* gene, and columns represent patients. As the gene expression of *F5* rose, the death number of the high-expression group increased, while the OS decreased. (d) Receiver operating characteristic curve was used to predict overall survival in gastric cancer patients based on *F5* expression level. TCGA: The Cancer Genome Atlas; *F5*: Coagulation factor V.

The baseline information of 351 GC patients is listed in Table 1. The clinical features including age (*n* (<60) = 108 vs. *n* (≥60) = 240), TNM stage (*n* (stage I) = 47 vs. *n* (stage II) = 109 vs. *n* (stage III) = 147 vs. *n* (stage IV = 35)), Pathological M (*n* (M1) = 312 vs. *n* (M0) = 24), Pathological T (*n* (T1/2) = 91 vs. *n* (T3/4) = 256), cancer status (*n* (no) = 206 vs. *n* (yes) = 118), radiation therapy (*n* (yes) = 62 vs. *n* (no) = 266) and targeted molecular therapy (*n* (yes) = 151 vs. *n* (no) = 175) were obtained and included in the multivariate Cox regression model after univariate survival analysis (Table 1). It was found that the high expression of the *F5* gene was significantly correlated with the shorter survival rate of GC patients, poor prognosis (high vs. low; median survival time (MST): 22 months vs. 38 months), and high risk of death (crude *P* = 0.024, crude HR (95%CI) = 1.457 (1.048, 2.025), Table 1, Figure 2; adjusted *P* = 0.036, adjusted HR (95%CI) = 1.533 (1.029, 2.284), Table 2)). The results of gene expression and survival time of the patients and the expression heat map of *F5* gene are presented in Figures 3(a)–3(c). The stratified results are shown in Table 3. Compared with the low-expression group, the high-expression group, comprising patients with pathological T3/4 (adjusted *P* = 0.03), pathological M0 (adjusted *P* = 0.049), stage IV (adjusted *P* = 0.031), GC patients treated with radiation therapy (adjusted *P* = 0.016), targeted molecular therapy (adjusted *P* = 0.03) and cancer-free survival (adjusted *P* = 0.007), had poorer prognosis. Notably, combining these clinical features and *F5* improved the prognostic accuracy for GC OS. Time-dependent ROC curves were plotted to assess the prediction accuracy of *F5* gene for prognosis of GC patients. The AUC of the time-dependent ROC curve at 1, 2, 5-year survival was medium as shown in Figure 3(d) (AUC: 0.554, 0.593, and 0.603, respectively).

### 3.3. KM-Plotter Survival Analyses

**Figure 4:**
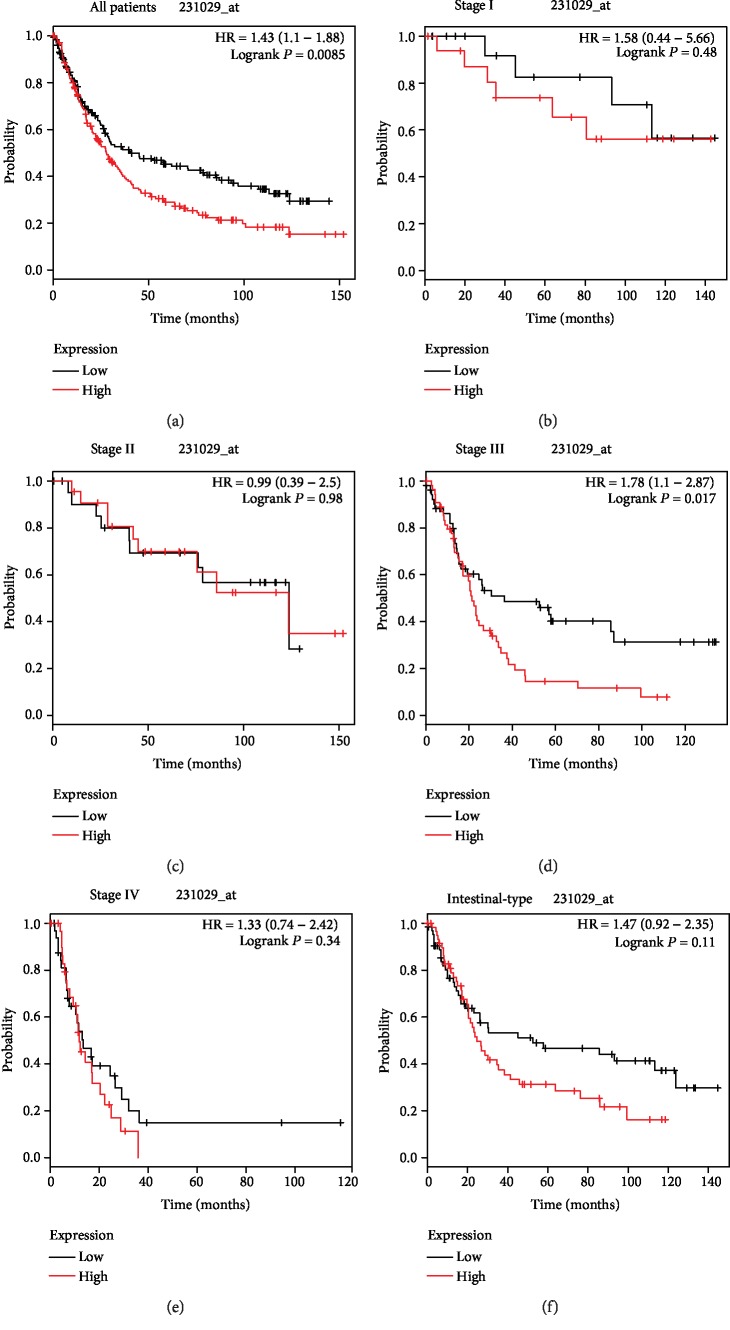
Prognostic value of the *F5* (231029_at) expression determined by the Kaplan-Meier plotter tool. Overall survival curves for (a) all patients and patients in (b) stage I, (c) stage II, (d) stage III, (e) and stage IV, and (f) their intestinal type. *F5*: Coagulation factor V.

**Figure 5:**
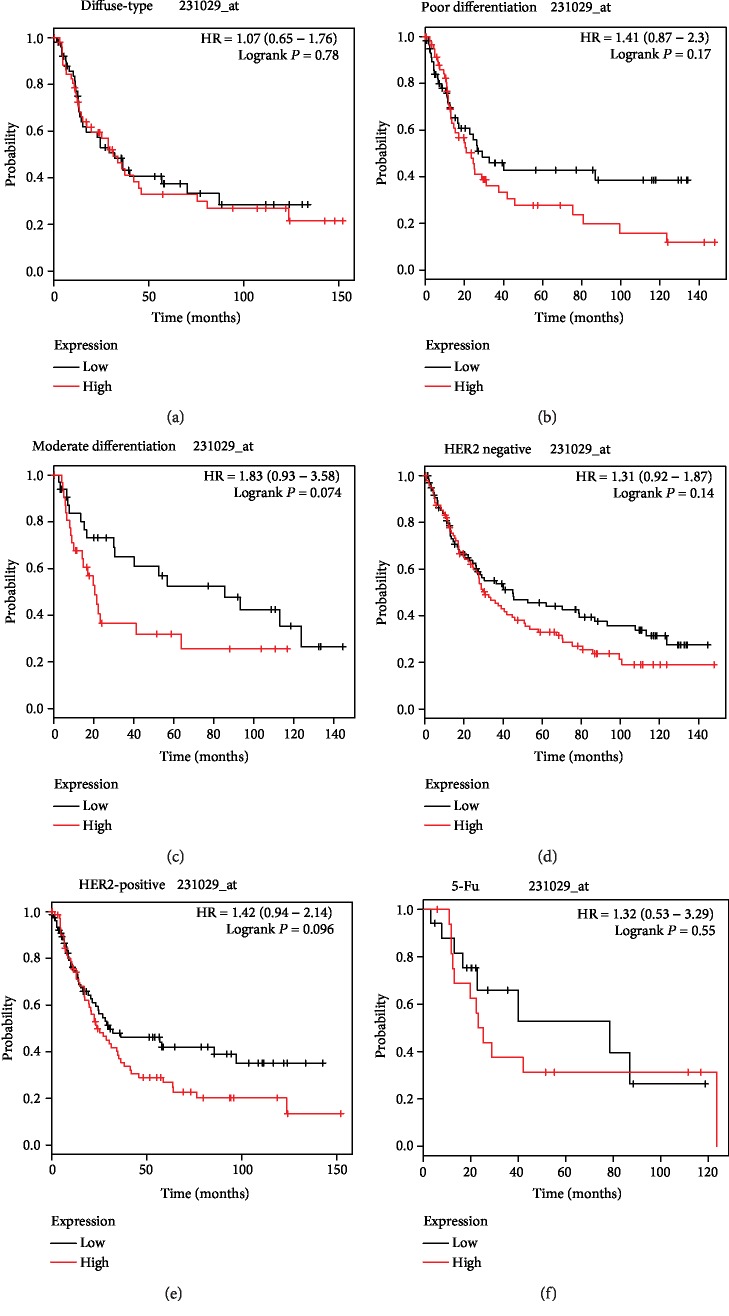
Prognostic value of the *F5* (231029_at) expression determined by the Kaplan-Meier plotter tool. Overall survival curves for (a) diffuse-type, (b) poor differentiation, (c) moderate differentiation, (d) HER2-negative, (e) HER2-positive, and (f) 5-Fu treatment gastric cancer. *F5*: Coagulation factor V.

Subsequently, the *F5* gene was submitted to the KM-plotter online website. The affymetrix ID 231029_at was employed to further explore the prognostic potential of the *F5* gene by assessing its correlation with various clinical variables of GC patients. As shown in Figure 4 and Table 4, a favourable OS was observed in all GC patients with a high expression of the *F5* gene (HR (95%CI) = 1.43 (1.1 − 1.88), *P* = 0.0085, Figure 4(a)). GC samples were subcategorized according to the variables for survival analysis. Briefly, samples were divided into TNM stage, Lauren classification, differentiation, etc. It was found that the *F5* gene could predict the prognosis of subjects in stage III (HR (95%CI) = 1.78 (1.1 − 2.87), *P* = 0.017, Figure 4(d)). However, the remaining clinical features had no statistical significance on OS between the high *F5* gene expression and low-expression groups (Figures 4(b), 4(c), 4(e), and 4(f) and Figures 5(a)–5(f)).

### 3.4. Genome-Wide Coexpression Analysis Result

**Figure 6:**
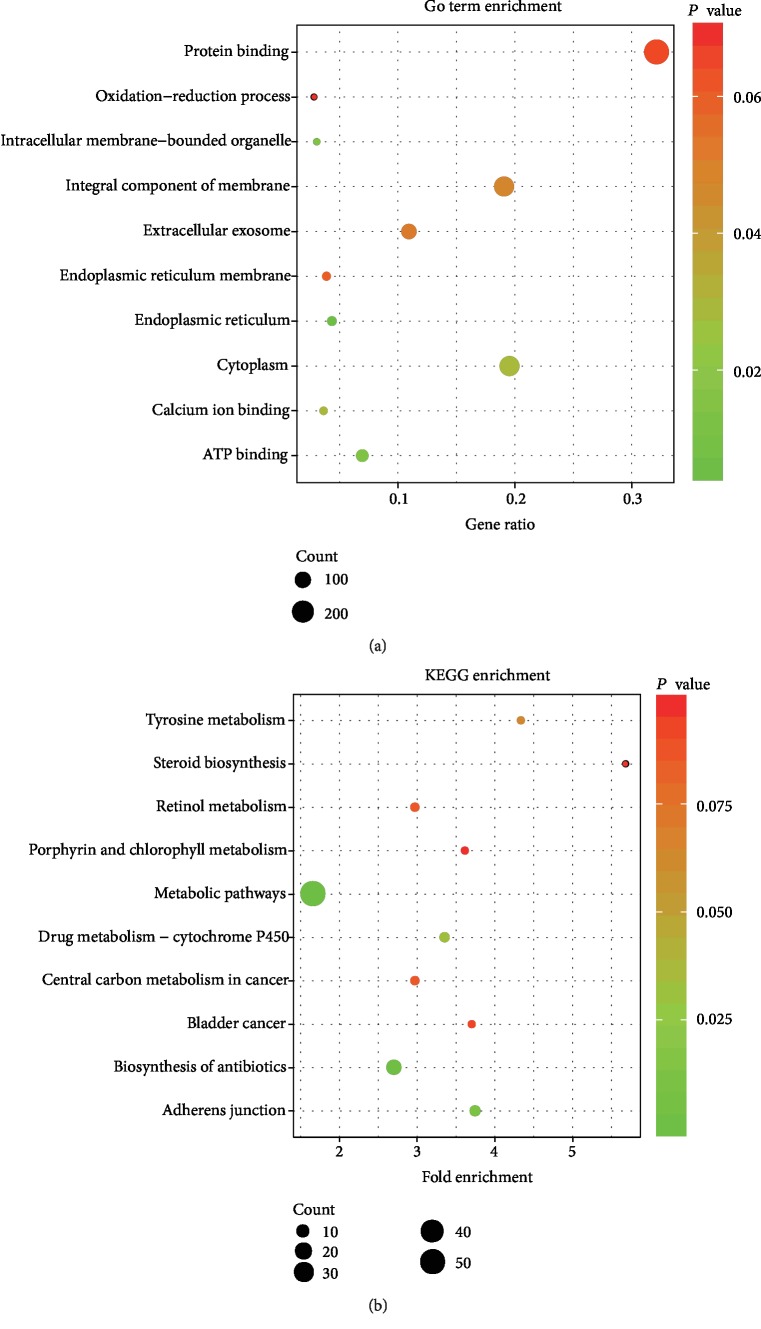
GO and KEGG analysis of genes coexpressed with *F5*. (a) GO enrichment analysis. (b) KEGG enrichment analysis. The “count” represents the number of genes significantly enriched in GO and KEGG. *F5*: coagulation factor V; GO: Gene Ontology; KEGG: Kyoto Encyclopedia of Genes and Genomes.

A total of 590 coexpressed genes were identified by coexpression analysis, and the results of the enrichment analysis are presented in Figure 6. Notably, the GO analysis indicated that coexpression of the *F5* gene was mainly enriched in protein binding, cytoplasm, integral component of the membrane, extracellular exosome, ATP binding, and oxidation-reduction process (Figure 6(a)). KEGG analysis revealed that the *F5* gene was enriched in pathways such as metabolic pathways, biosynthesis of antibiotics, drug metabolism-cytochrome P450, and adherens junction (Figure 6(b)).

### 3.5. GSEA

**Figure 7:**
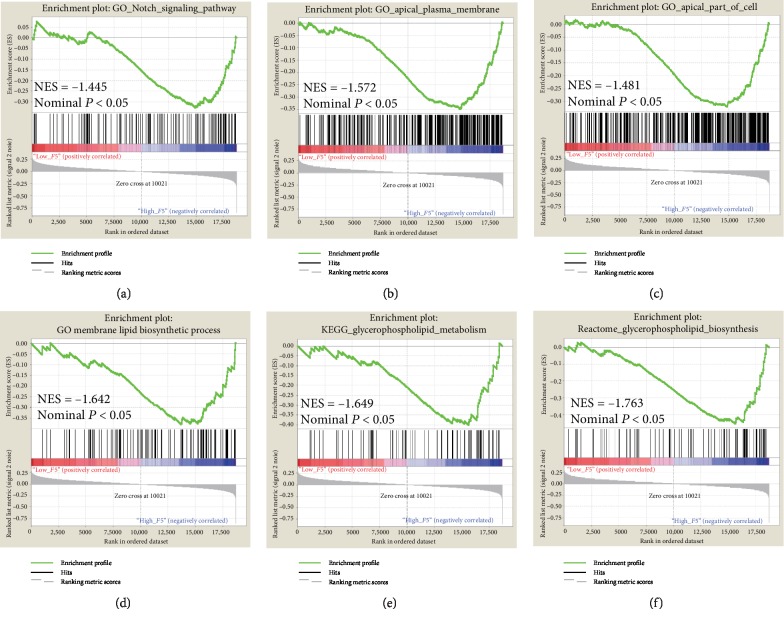
GSEA analysis of *F5* in gastric cancer patients using TCGA database. The GO terms including (a) “Notch signaling pathway,” (b) “apical plasma membrane,” (c) “apical part of cell,” and (d) “membrane lipid biosynthetic process” and the KEGG terms including (e) “reactome glycerophospholipid biosynthesis” and (f) “glycerophospholipid metabolism” were enriched in the *F5* high-expression phenotype. *F5*: Coagulation factor V; GO: Gene Ontology; KEGG: Kyoto Encyclopedia of Genes and Genomes; ES: enrichment score; GSEA: gene set enrichment analysis; NES: normalized enrichment score.

The results of single gene set enrichment analysis are shown in Figure 7. In the c5 category, the *F5* gene was enriched in the Notch signalling pathway (Figure 7(a)), apical plasma membrane (Figure 7(b)), apical part of cell (Figure 7(c)), and membrane lipid biosynthetic process (Figure 7(d)) in the GO dataset, while in the c2 category, the *F5* gene was associated with reactome glycerophospholipid biosynthesis (Figure 7(e)) and glycerophospholipid metabolism (Figure 7(f)) in the KEGG dataset. This indicates that the potential mechanisms of *F5* are likely mediated through their influence on the tight junction among cells and the glycerophospholipid metabolism pathway.

## 4. Discussion

Previous studies have reported that cancer-induced hemostatic activity promotes tumour growth and metastasis in patients. It has also been demonstrated that tissue factor (TF) regulates VEGF synthesis and enhances its level in tumour cells [4, 18] [19]. Activation of *F5*, a Janus-faced protein in the coagulation cascade, not only promotes the production of thrombin as the cofactor of FXa but also inactivates FVIIIa and FVa as anticoagulant cofactors of the activated protein C (APC) [3, 20]. So far, few studies have explored the prognostic value of the *F5* gene expression in cancer patients. This study reveals that the high expression of *F5* in gastric cancer predicts poor survival time.

*F5* has been found to be an oncogene. Tinholt et al. analysed a cohort of 1100 breast cancer samples from TCGA and found that the expression of *F5* mRNA was about 2-fold higher in breast tumours compared to normal tissues, and its expression increased in patients with late stage tumours [7]. Klee et al. also found that the *F5* gene was upregulated in cancer tissue compared to nonneoplastic prostate tissue [21]. Here, we show that *F5* is highly expressed in gastric cancer tissue and this strongly correlates with advanced TNM stage and shorter OS. The ROC curve reveals that the *F5* gene can distinguish tumour from normal tissues with an AUC of 0.705. These findings indicate that *F5* may be a possible therapeutic target for GC.

A couple of previous studies have reported the *F5* gene as a risk marker of cancer. For instance, Vossen et al. demonstrated that the *F5* gene polymorphism is associated with the susceptibility to colorectal cancer in a German population compared with controls, and similar results were obtained for breast cancer [22, 23]. However, other researchers found that *F5* is not a risk factor for cancers such as gynaecological and oral cancers and gliomas [24–28]. Thus, it is likely that *F5* may contribute to tumourigenesis. Interestingly, our study shows that the high expression of *F5* is correlated with a poor OS in GC, and this contradicts a previous study in which high *F5* expression improved OS for the basal type of tumours in breast cancer according to the KM-plotter website [7]. However, this could have been caused by failure to exclude other confounding factors and the use of only univariate survival analysis in the study. A comprehensive analysis of *F5* in gastric cancer is therefore advocated. Further analysis using the KM-plotter survival analysis was performed revealing that the high expression of *F5* correlated with shorter OS for all GC samples and stage III GC. Remarkably, no statistical significance is observed in the rest of the clinical features in the KM-plotter database, but the HR of these variables is higher than one, indicating a high risk of death. For patients in stage III, the *P* value of survival analysis determined by KM-plotter was less than 0.05, but not for TCGA. Yet, the overall risk of stage III patients from TCGA was also higher than one (HR = 1.318), and it is similar to that from the KM-plotter website (HR = 1.78). As the sample size increases, the *P* value may be less than 0.05. Hence, this needs further clarification through large scale clinical trials. The time-dependent ROC revealed that *F5* could predict the OS in GC. These findings show that *F5* may be an independent prognostic factor that negatively predicts survival time in GC patients.

The GSEA is a platform used to identify biological processes. Here, GSEA analysis showed that the high expression of *F5* was related to the activation of the Notch signalling pathway and the promotion of membrane lipid biosynthetic process, apical plasma membrane, apical junction complex, reactome glycerophospholipid biosynthesis, and glycerophospholipid metabolism. Genome-wide coexpression analysis indicated that the coexpressed genes play important roles in an integral component of the membrane and metabolic pathways. Several studies have demonstrated the relationship between these biological mechanisms and cancer prognosis. A previous meta-analysis has implicated the participation of the activated Notch signalling pathway in the progression of gastric cancer [29]. Li et al. also showed that the activation of the Notch signalling pathway regulates the development and progression of gastric cancer [30]. Tight junctions (TJ) located between cells play important roles in paracellular solute transport and cell polarity maintenance. Therefore, defects in TJ structure and function trigger cancer initiation and development [31]. Our results show that the *F5* gene is enriched in tight junction-related pathways, such as the apical junction complex. It is well known that the fatty acid metabolism pathway contributes to the development of cancer [32]. Shu et al. found that abnormal metabolic regulation of glycerophospholipids is associated with the pathogenesis of colorectal cancer [33]. Inhibition of glycerophospholipid biosynthesis by a key enzyme of lysophosphatidic acid acyltransferase *β* (LPAAT-*β*) may be a potential therapeutic target for osteosarcoma patients [34]. Henderson et al. found that glycerophospholipid metabolism is enhanced in melanocyte neoplasia in zebrafish where it accelerates tumour progression [35]. Enrichment analysis results show that biosynthesis and metabolism of glycerophospholipids are among the biological pathways involved in cancer progression. Therefore, we speculate that *F5*, as a potential oncogene, may affect the prognosis of cancer patients through these biological pathways and overexpression of *F5* would lead to poor prognosis in GC.

The current study has some limitations. Firstly, the outcomes of survival analysis need further verification because all the research data used were from an open database. Secondly, the mechanism of *F5* regulation in tumourigenesis and the progression of gastric cancer were not further explored. Thirdly, the KM plotter data was from multiple databases and the samples were probably collected at different places using different protocols. Fourthly, as TCGA cohort was unable to obtain the clinical information of postoperative chemotherapy of gastric cancer patients, we could not find the relationship between the expression level of the *F5* gene and the prognosis of gastric cancer patients receiving postoperative chemotherapy. Meanwhile, as only 34 gastric cancer patients treated with postoperative chemotherapy were provided on the KM-plotter website and the sample size was small, the significant correlation between the expression level of *F5* and the prognosis of gastric cancer patients treated with 5-FU-based chemotherapy could not be observed.

Despite these limitations, this is the first study to reveal the association of *F5* mRNA expression with the clinical outcome of GC patients. Univariate and multivariate survival analyses reveal that *F5* is an independent prognostic factor for OS of GC patients. This conclusion was verified on the KM-plotter website. Thus, *F5* may serve as a potential therapeutic target in GC. The genome-wide coexpression and GSEA analysis were also used to reveal the biological pathways that underlie the prognostic role of the *F5* gene for OS in GC patients, which can provide guidance for the exploration of its mechanism in the future. Once these results are confirmed, we anticipate that *F5* will be applied in clinical settings to monitor the prognosis and develop management and therapeutic strategies for GC.

## 5. Conclusions

In conclusion, the current findings show that the *F5* gene is upregulated in GC tumour tissues and may be a potential prognostic biomarker for GC. However, these results require further verification.

## Figures and Tables

**Table 1:** Correlation between OS and clinicopathologic features of GC patients.

Variables	Events/total (*n* = 351)	MST (months)	HR (95% CI)	Logrank *P* value
*F5*				
Low	64/176	38	1	
High	80/175	22	1.457 (1.048, 2.025)	0.024
Missing	0			
Age (years)				
<60	36/108	60	1	
≥60	108/240	26	1.549 (1.061, 2.263)	0.022
Missing	3			
Gender				
Female	100/226	29	1	
Male	44/125	35	0.784 (0.55, 1.118)	0.178
Missing	0			
Pathological T				
T 1/2	28/91	70	1	
T 3/4	112/256	26	1.730 (1.138, 2.269)	0.009
Missing	4			
Pathological N				
N0/N1	70/168	29	1	
N2/N3	69/173	29	0.996 (0.714, 1.390)	0.98
Missing	10			
Pathological M				
M0	13/24	13	1	
M1	125/312	35	1.955 (1.103, 3.467)	0.019
Missing	15			
TNM stage				
Stage I	11/47	73	1	
Stage II	34/109	56	1.608 (0.813, 3.182)	<0.001
Stage III	69/147	26	2.435 (1.286, 4.61)	
Stage IV	22/35	16	3.789 (1.836, 7.819)	
Missing	13			
Histologic grade				
G1/G2	50/136	43	1	
G3	90/206	26	1.366 (0.966, 1.932)	0.077
Missing	9			
Radiation therapy				
Yes	19/62	NA	1	
No	116/266	26	2.322 (1.418, 3.802)	0.001
Missing	23			
Targeted molecular therapy				
Yes	56/151	43	1	
No	78/175	26	1.489 (1.055, 2.1)	0.022
Missing	25			
Tumour location				
Cardia	36/84	26	1	
Body	50/123	28	0.916 (0.596, 1.407)	0.919
Antrum	52/130	35	0.936 (0.611, 1.432)	
Missing	14			
Histology				
Intestinal	64/160	38	1	
Diffuse	24/61	60	1.001 (0.625, 1.603)	0.057
Signet ring cell	8/11	13	2.515 (1.204, 5.251)	
Others	48/118	26	1.287 (0.884, 1.875)	
Missing	1			
MSI status				
MSS	99/240	28	1	
MSI-L	22/51	29	1.263 (0.794, 2.007)	0.225
MSI-H	23/59	35	0.756 (0.48, 1.191)	
Missing	1			
Cancer status				
No	35/206	NA	1	
Yes	86/118	17	5.526 (3.695, 8.264)	<0.001
Missing	27			

Abbreviations: *F5*—Coagulation factor V; HR—hazard ratio; MST—median survival time; OS—overall survival; GC—gastric cancer.

**Table 2:** Multivariate analyses of *F5* in the prediction of gastric cancer overall survival.

Variables	HR (95% CI)	*P* value
*F5* (high vs. low)	1.533 (1.029, 2.284)	0.036
Age (years) (≥60 vs. <60)	1.581 (1.029, 2.429)	0.037
Pathological M (M1 vs. M0)	1.129 (0.407, 3.132)	0.816
Pathological T (T3/4 vs. T1/2)	1.051 (0.559, 1.978)	0.877
TNM stage		
Stage II vs. stage I	2.058 (0.727, 5.830)	0.174
Stage III vs. stage I	2.562 (0.895, 7.330)	0.079
Stage IV vs. stage I	3.014 (0.931, 9.758)	0.066
Radiation therapy (no vs. yes)	1.467 (0.771, 2.791)	0.243
Targeted molecular therapy (no vs. yes)	1.367 (0.861, 2.170)	0.185
Cancer status (yes vs. no)	5.193 (3.296, 8.183)	<0.001

Abbreviations: *F5*—Coagulation factor V; HR—hazard ratio; CI—confidence interval; T—tumour; N—node; M—metastasis.

**Table 3:** Stratified analysis of *F5* genes and OS in GC patients.

Group	Low	High	Crude HR (95% CI)	Crude *P* value	Adjusted HR (95% CI)	Adjusted *P* value^a^
Age (years)						
<60	53	55	1.315 (0.68, 2.545)	0.416	1.878 (0.883, 3.994)	0.102
≥60	121	119	1.459 (0.996, 2.136)	0.052	1.446 (0.949, 2.202)	0.086
Pathological T						
T 1/2	52	39	1.332 (0.632, 2.805)	0.451	1.164 (0.483, 2.806)	0.735
T 3/4	120	136	1.39 (0.96, 2.01)	0.81	1.56 (1.043, 2.334)	0.03
Pathological M						
M0	11	13	0.418 (0.128, 1.37)	0.15	13.597 (1.012, 182.75)	0.049
M1	159	153	1.373 (0.965, 1.953)	0.078	1.357 (0.925, 1.991)	0.119
TNM stage						
Stage I	28	19	1.726 (0.51, 5.839)	0.38	7.483 (0.732, 76.529)	0.09
Stage II	59	50	1.28 (0.652, 2.513)	0.474	1.235 (0.625, 2.438)	0.544
Stage III	68	79	1.439 (0.891, 2.324)	0.137	1.318 (0.798, 2.177)	0.281
Stage IV	14	21	1.852 (0.71, 4.83)	0.207	3.856 (1.135, 13.101)	0.031
Radiation therapy						
Yes	30	32	3.389 (1.207, 9.522)	0.021	4.607 (1.323, 16.035)	0.016
No	137	129	1.282 (0.89, 1.847)	0.182	1.345 (0.913, 1.981)	0.133
Targeted molecular therapy						
Yes	85	66	1.558 (0.919, 2.641)	0.099	1.885 (1.065, 3.338)	0.03
No	82	93	1.3 (0.829, 2.04)	0.254	1.35 (0.839, 2.17)	0.216
Cancer status						
No	108	98	2.515 (1.25, 5.061)	0.01	2.852 (1.327, 6.127)	0.007
Yes	54	64	1.366 (0.884, 2.112)	0.161	1.277 (0.801, 2.037)	0.304

^a^Adjusted for age, TNM stage, radiation therapy, and targeted molecular therapy; the missing patients of these clinical parameters are the same as Table 1. Abbreviations: *F5*—Coagulation factor V; HR—hazard ratio; OS—overall survival; GC—gastric cancer.

**Table 4:** The prognostic value of the mRNA expression of *F5* in various clinical features GC patients from KM-plotter.

Group	Low	High	HR (95% CI)	*P* value
All	177	171	1.43 (1.1−1.88)	0.0085
Histology				
Intestinal	65	63	1.47 (0.92−2.35)	0.11
Diffuse	52	53	1.07 (0.65−1.76)	0.78
Mixed	11	11	1.84 (0.45−7.44)	0.39
TNM stage				
Stage I	17	17	1.58 (0.44−5.66)	0.48
Stage II	22	22	0.99 (0.39−2.5)	0.98
Stage III	54	55	1.78 (1.1−2.87)	0.017
Stage IV	34	32	1.33 (0.74−2.42)	0.34
HER2 status				
Negative	99	96	1.31 (0.92−1.87)	0.14
Positive	78	75	1.42 (0.94−2.14)	0.096
Differentiation				
Poorly	60	61	1.41 (0.87−2.3)	0.17
Moderately	34	33	1.83 (0.93−3.58)	0.074
Well	2	3		
5-Fu treatment	17	17	1.32 (0.53–3.29)	0.55

Abbreviations: *F5*—Coagulation factor V; HR—hazard ratio; OS—overall survival; GC—gastric cancer, T—tumour; N—node; M—metastasis.

## Data Availability

The data used to support the findings of this study are available from the corresponding author.
